# Terahertz high-resolution spectroscopy of thermal decomposition gas products of diabetic and non-diabetic blood plasma and kidney tissue pellets

**DOI:** 10.1117/1.JBO.26.4.043008

**Published:** 2021-03-08

**Authors:** Anastasiya A. Lykina, Vladimir A. Anfertev, Elena G. Domracheva, Mariya B. Chernyaeva, Yulia A. Kononova, Yana G. Toropova, Dmitry V. Korolev, Olga A. Smolyanskaya, Vladimir L. Vaks

**Affiliations:** a ITMO University, Institute of Photonics and Optical Information Technologies, Saint Petersburg, Russia; b Institute for Physics of Microstructures, Russian Academy of Sciences, Nizhny Novgorod, Russia; c Lobachevsky State University, Nizhny Novgorod, Russia; d Almazov National Medical Research Centre, Saint Petersburg, Russia

**Keywords:** high-resolution terahertz spectroscopy, thermal decomposition, tissue, blood plasma, diabetes, lyophilization

## Abstract

**Significance**. One of the modern trends in medical diagnostics is based on metabolomics, an approach allowing determination of metabolites which can be the specific features of disease. High-resolution gas spectroscopy allows investigation of the gas metabolite content of samples of biological origin. We present the elaboration of a method of studying diabetic and non-diabetic biological samples, prepared as pellets, by terahertz (THz) high-resolution spectroscopy.

**Aim:** The main idea of the work is studying the content of thermal decomposition gas products of diabetic and non-diabetic dried blood plasma and kidney tissues for revealing the set of gas-markers that characterized the diabetes by the THz high-resolution spectroscopy method.

**Approach:** We present an approach to study the diabetic and non-diabetic blood plasma (human and rats) and kidney tissues (rats), using high-resolution spectroscopy based on the non-stationary effect of THz frequency range. The methods of preparing the blood and kidney tissue samples as pellets and of vaporizing the samples were developed.

**Results:** The measurements of rotational absorption spectra of vapors at heating the pellets prepared from blood and kidney tissue were carried out in 118 to 178 GHz frequency range. The absorption lines appearing in spectra of the sample vapors were detected and identified. The molecular contents of thermal decomposition products differed for non-diabetic and diabetic samples; e.g., main marker is acetone appearing in the diabetic blood (human and rats) and in the diabetic kidney tissue.

**Conclusions:** Our paper illustrates the potential ability for determining the metabolite content of biological samples for diagnostics and prognosis of diseases for clinical medicine.

## 1 Introduction

Nowadays, the problem of diagnostics of socially important diseases (diabetes, cancer diseases, etc.), including the early stage, attracts the great attention of scientific groups in the world. One of the new approaches opening new possibilities in diagnostics and treatment of various diseases is metabolomics. Metabolomics is a field of science that investigates the final and intermediate metabolic products of a biological system (cell, organs, or whole organism). The metabolomics can reveal the metabolites which can be the specific features of disease and can play a large role in diagnosis and prognosis for a disease. The media, containing the final and intermediate metabolic products in the human organism and useful for revealing the metabolites associated with diseases and pathologies, are exhaled breath, blood, urine, saliva etc.

One of the socially important diseases threatening to the quality of life of people is diabetes. Diabetes mellitus type 2 was previously a disease of middle age, but now it is diagnosed in adults, young people, and children. The results of metabolomic investigations of blood and urine samples of diabetes patients from 18 years as well as children from 1 to 13 years old are presented in Human Metabolome Database (HMDB).^1^ Mainly, blood (21 chemical substances including isomers), urine (30 chemical substances including isomers), and cerebrospinal fluid (1 chemical substance) were investigated. For example, (R)-3-hydroxybutyric acid was detected in all three types of fluids. Some substances (acetoacetic acid, D-fructose, and D-glucose) were detected in both liquids (blood and urine). The more characteristic feature of diabetes (acetone, ${\mathrm{CH}}_{3}{\mathrm{COCH}}_{3}$) was present for urine only. The liquids samples, the results of the investigations presented in HMDB, have been investigated by various methods—gas chromatography;^2^ gas chromatography with mass-spectrometry;^3^^,^^4^ gas-liquid chromatography with mass-spectrometry;^5^ high-resolution spectroscopy of nuclear proton magnetic resonance;^6^ and methods using the special devices for diabetes patient’s state monitoring, the continuous glucose monitoring system.^7^

The development of chronic kidney disease is a major complication of diabetes mellitus. Although the determination of glycated albumin is the common approach to the screening, kidney damage may start long before clinically significant changes in urine albumin appear. Considering the multifactorial pathogenesis of chronic kidney disease, various markers have been considered for its screening.^8^^,^^9^ However, their diagnostic value without kidney biopsy appeared to be doubtful.

The spectroscopic approach can provide the information about content of the biological samples which can be used for medicine diagnostics. The terahertz (THz) frequency range is very important for studying gases, liquids, and tissues of living organisms. There are some reviews devoted to applications of methods of THz frequency range, including THz time-domain spectroscopy, THz reflectometry, and THz imaging for biology and medicine.^10^

THz time-domain spectroscopy allows detecting the spectra, where the absorption coefficient or refraction index has some spectral features corresponding the presence of some biomolecules (proteins, sugars, etc.) in the samples or their various concentrations.^11^

The molecular absorption spectroscopy and in particular THz gas spectroscopy is a very promising approach for investigations of multicomponent gas mixtures of various origins.^12^ The samples in liquid and solid state can be investigated by vaporing or thermal decomposition. The metabolites-markers of socially important diseases (diabetes, cancer, etc.) can be revealed by its spectral lines in the absorption spectra carried out at transmission of radiation through gas sample.

The level of acetone in samples of exhaled breath and urine taken from the diabetes patients (12 people) was measured using a terahertz range spectrometer with phase-shift keying of the acting radiation. Experiments were done in collaboration with Almazov National Medical Research Centre. The measurements were carried out at the acetone absorption lines with central frequencies of 150.537 and 151.647 GHz. The measurements were carried out without heating. Simultaneous analysis of the samples of exhaled breath and urine of diabetes patients revealed that the acetone level in the urine was much higher than in the exhaled breath, in some cases an order of magnitude higher.^13^^,^^14^

The results of THz high-resolution gas spectroscopy investigations of blood plasma of patients and rats (conditionally healthy and with diabetes) as well as the rat’s kidney tissue (healthy and with diabetes) dried and pressed to pellets are presented. The tissues or blood plasma of living organisms, without any preparation, must be investigated immediately after sampling. Preparation as pellets allows investigation after a sufficiently long time after blood sampling or kidney biopsy.

## 2 Experiments and Methodology

### 2.1 Method for the Preparation of Blood Plasma Pellets and Kidney Pellets

Venous blood from patients with type 2 diabetes mellitus and conditionally healthy participants were collected at the Endocrinology Department of Almazov National Medical Research Centre; the center provides medical care for diabetic patients. Three patients and two participants were male, age-matched (39 to 43 years old). All experimental protocols used in this investigation were reviewed and approved by the patients and participants and the Use Commission of the Medical Centre. Venous blood was collected in the morning after 8 to 12 hours of fasting in a tube with the anticoagulant $K_{3}\mathrm{EDTA}$ (Vacutest Kima, Italy). Plasma was obtained for the analysis of biochemical parameters by centrifugation of whole blood at 3000 rpm for 15 min in a laboratory centrifuge (Eppendorf 5702R, Germany) at a temperature of +4°C. Values of biochemical parameters of blood plasma samples and reference intervals are presented in Table 1 (the level of glycated hemoglobin was obtained on the whole blood). Table 1 shows that the concentration of glucose, triglycerides, and glycated hemoglobin in the samples of a patient with diabetes increases 1.5, 2, and 2.3 times, respectively.

**Table 1:** Biochemical parameters levels in human blood plasma samples.

Biochemical parameter	Healthy sample	Diabetic sample	Reference interval
Albumin (g/l)	49.10	45.20	34 to 48
Glucose (mmol/l)	4.34	6.51	3.30 to 6.10
Triglycerides (mmol/l)	0.82	1.69	<1.77
Glycated hemoglobin (%)	4.8	11.0	4 to 6
Bilirubin (mmol/l)	0.027	0.007	0.003 to 0.020
Creatinine (mmol/l	0.08	0.06	0.06 to 0.10
Total cholesterol (mmol/l)	4.49	3.43	3.50 to 5.00
Uric acid (mmol/l)	0.25	0.15	0.20 to 0.42

The study was also carried out with Wistar male rats at the age of 8 weeks and weight 180 to 200 g, according to the protocol of experimental studies approved by the Animal Care and Use Commission of the Institute of Experimental Medicine Almazov National Medical Research Centre. The glucose level in animals of the experimental group was 21  mmol/l after 120 min after glucose loading. Intact rats were used as control group. Venous blood was collected from the inferior vena cava before animals were euthanized. Also, the kidney was harvested. Blood samples were collected into tubes containing an anticoagulant.

Test samples were frozen at a temperature of −80°C (low-temperature refrigerator DW-86L388A, Haier, China). Then it was lyophilized by freeze-drying VaCo 2 (ZirBus, Germany) at a temperature of −50°C and a pressure of 3 Pa. Freezing is carried out before lyophilization, since during lyophilization under the influence of high internal pressure, biological components can be destroyed. Dried samples were a sponge consisting of biological crystals. The sponge was destroyed by a metal spatula and crushed to crystals with a size of several tens of micrometers. The use of a mortar and pestle was impossible, since grinding the proteins in the composition of the samples would lead to their unwanted adhesion and the formation of round granules.

The lyophilized samples powder was weighed (analytical balance OHAUS Discovery, Switzerland) and then placed in a steel press-mold. Using a laboratory presses (Enkor, Russia and Specac, UK) at a certain molding pressure, the blood plasma pellets and kidney pellets were obtained. Each crystal of pellets contains a certain percentage of fats (triglycerides), proteins (albumin), and fibrinogen—all of them normal or glycated (in the diabetic case). The pellets of samples from the control group hereinafter are referred as “non-diabetic pellet” and the pellets of samples from the diabetic group hereinafter are referred as “diabetic pellet.”

### 2.2 THz High-Resolution Spectroscopy Experimental Setup

High-resolution THz spectroscopy based on non-stationary effects allows analyzing the component composition of gas and vapor multicomponent gas mixtures. Spectrometers provide sensitivity close to the theoretical limit with a resolution limited only by the Doppler effect and can record fast processes. The sensitivity is preserved even with a substantial decrease in gas pressure and is approximately 0.2 ppb in the scanning mode for some gases (e.g., ammonia at the measurements of absorption line near the frequency of 572 GHz). In addition, it enables determining the component composition of a gas mixture with high reliability.^12^

A study of the thermal decomposition products of pellets was carried out using the method of THz non-stationary gas spectroscopy of the 118 to 178 GHz range. The spectrometer uses a phase-locked loop for automatic control of the frequency of the backward wave lamp and phase shift, registration in the time-domain, averaging, and processing of the spectroscopic transition signal. The device registers a signal in the time domain. Samples’ vapor was analyzed using the following procedure. Pellets were placed into a test tube, using vacuum pumped, gradually heated a test tube (up to 240°C) and the resulting mixture of thermal decomposition products of the sample was let into the measuring cell. A data file usually includes recordings of a selected or of the total operating range of the spectrometer. Therefore, substances can be identified by absorption lines appearing in the total spectrum of the sample and the dynamics of concentrations in a mixture can be traced by comparing several data files. Substances were identified by searching them in open-source mw, mmw, and THz databases.^15^^,^^16^

## 3 Blood Plasma Vapors and Kidney Vapors Analysis with Using High-Resolution Terahertz Spectrometer

**Fig. 1:**
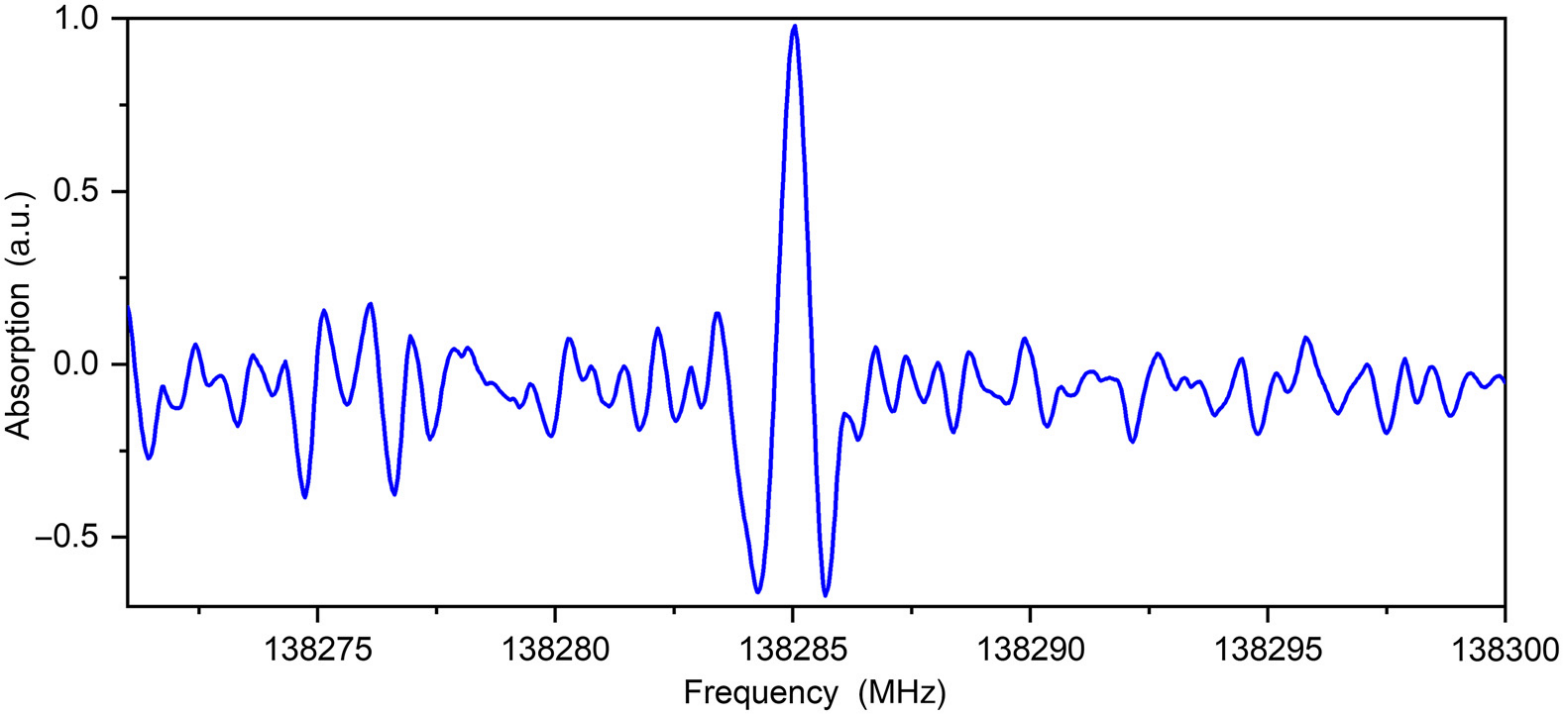
Recording parts of the absorption spectra with a line of acetone (f=138.28528  GHz, lgI=−5.5867, 38 28 10 0 ← 38 27 11 1) in a sample of diabetic human blood plasma in THz spectrometer operating range.

With our THz-TDS systems, we measured pellets transmission in the spectral range of 0.2 to 1.4 THz,^17^ but we know that biomolecules and in particularly its decomposition products in gas state have informative response (rotational absorption spectra) at these frequencies and lower also, thus we performed additional study on THz high-resolution gas spectrometer. The detected absorption lines of substances in diabetic human blood plasma coincide with substances of non-diabetic human blood plasma, except for acetone. There are many absorption lines of acetone lying in the working frequency range of spectrometer (118 to 178 GHz). Usually, it is more than enough to detect some absorption line of one substance for unambiguous determination of the presence of the substance in multicomponent gas mixture. Example of recording parts of the absorption spectra with the absorption line of acetone is shown in Fig. 1. Acetone can appear in the blood when carbohydrate metabolism disorder. As noted in the literature, the blood of patients with diabetes contained β-OH-butyrate and acetoacetate, but later these substances decomposed into acetone and carbon dioxide.^18^ Carbon dioxide has no dipole moment; therefore, it has no rotational transitions. Its increased content can be detected only by studying the vibrational spectrum of the sample in the IR range.

**Table 2:** Substances detected as a result of thermal decomposition of dry human blood plasma samples.

Substances	Central frequency of absorption line of dry blood plasma diabetic samples (GHz)	Central frequency of absorption line of dry blood plasma non-diabetic samples (GHz)	Central frequency of absorption line from databases^15^^,^^16^ (GHz)	*LgI*, where I (${\mathrm{nm}}^{2}\;\mathrm{MHz}$) from databases^15^^,^^16^	Quantum numbers of transition from databases^15^^,^^16^	State from databases^15^^,^^16^
Sulfur dioxide	142.0440	142.0440	142.0441	−5.2093	16 5 11 ← 17 4 14	v2
Ammonia	140.1420	140.1420	140.1418	−5.0383	2 1 2 ← 1 1 3	v2
Methanethiol	121.6837	121.6837	121.6835	−4.7691	15 1 14 1 ← 15 0 15 1	v=0 to 2
	124.0800	124.0800	124.07996	−4.7075	11 -1 11 0 ← 11 0 11 0	v=0 to 2
Carbonyl sulfide	121.7726	121.7726	121.7727	−4.4529	10-1 ← 9 1	v2=1
	121.625	121.625	121.6246	−3.3522	10 ← 9	Ground
Isocyanic acid	131.8856	131.8856	131.8849	−5.2238	6 0 6 5 ← 5 0 5 5	Ground
			131.88573	−3.6053	6 0 6 7 ← 5 0 5 6	Ground
			131.88574	−3.6797	6 0 6 6 ← 5 0 5 5	Ground
			131.88575	−3.7546	6 0 6 5 ← 5 0 5 4	Ground
			131.8864	−5.2238	6 0 6 6 ← 5 0 5 6	Ground
Acetone	125.1811	—	125.18107	−5.8058	8 5 4 0 ← 7 4 3 0	Ground
	138.285	—	138.28528	−5.5867	38 28 10 0 ← 38 27 11 1	Ground
	142.1153	—	142.1151915	−5.7786	22 6 16 0 ← 22 5 17 0	Ground
			142.1151916	−5.5567	22 7 16 0 ← 22 6 17 0	Ground
Formic acid	133.7671	133.7671	133.76707	−3.8331	6 0 6 ← 5 0 5	Ground

Plasma vapor substances, such as carbonyl sulfide (OCS), sulfur dioxide (${\mathrm{SO}}_{2}$), formic acid (HCOOH), isocyanic acid (HNCO), and ammonia (${\mathrm{NH}}_{3}$), were detected in both human diabetic and human non-diabetic samples. In some cases, there is one experimental line detected in spectra of the sample corresponding to more lines than one for one substance (e.g., isocyanic acid) in catalog data (see Table 2).^15^^,^^16^

This is explained by the fact that the catalogs contain data on the hyperfine structures of substances that are not resolved in this experiment as the lines have a finite width of the order of several hundred kHz. The obtained absorption lines of the studied substances coincide with the absorption spectra of vapors of capillary blood and blood plasma of patients with diabetes and conditionally healthy participant which was published in our previous work.^19^ The detected gaseous substances could have formed during sample preparation as products of chemical transformation of amino acids. The question of whether these compounds are of diagnostic value requires investigation. In clinical practice in diabetology, the analysis of the content of these substances is not used yet.

The vapors of blood plasma of healthy rat contained carbonyl sulfide (OCS), methanethiol (${\mathrm{CH}}_{3}\mathrm{SH}$), butyronitrile ($C_{3}H_{7}\mathrm{CN}$), acetaldehyde (${\mathrm{CH}}_{3}\mathrm{CHO}$), and formic acid (HCOOH). The sample of blood plasma of diabetic rat was differed by the acetone presence. In addition to the absorption lines of propyonitrile ($C_{2}H_{5}\mathrm{CN}$), methyl formate (${\mathrm{CH}}_{3}\mathrm{OCHO}$) was detected.

**Fig. 2:**
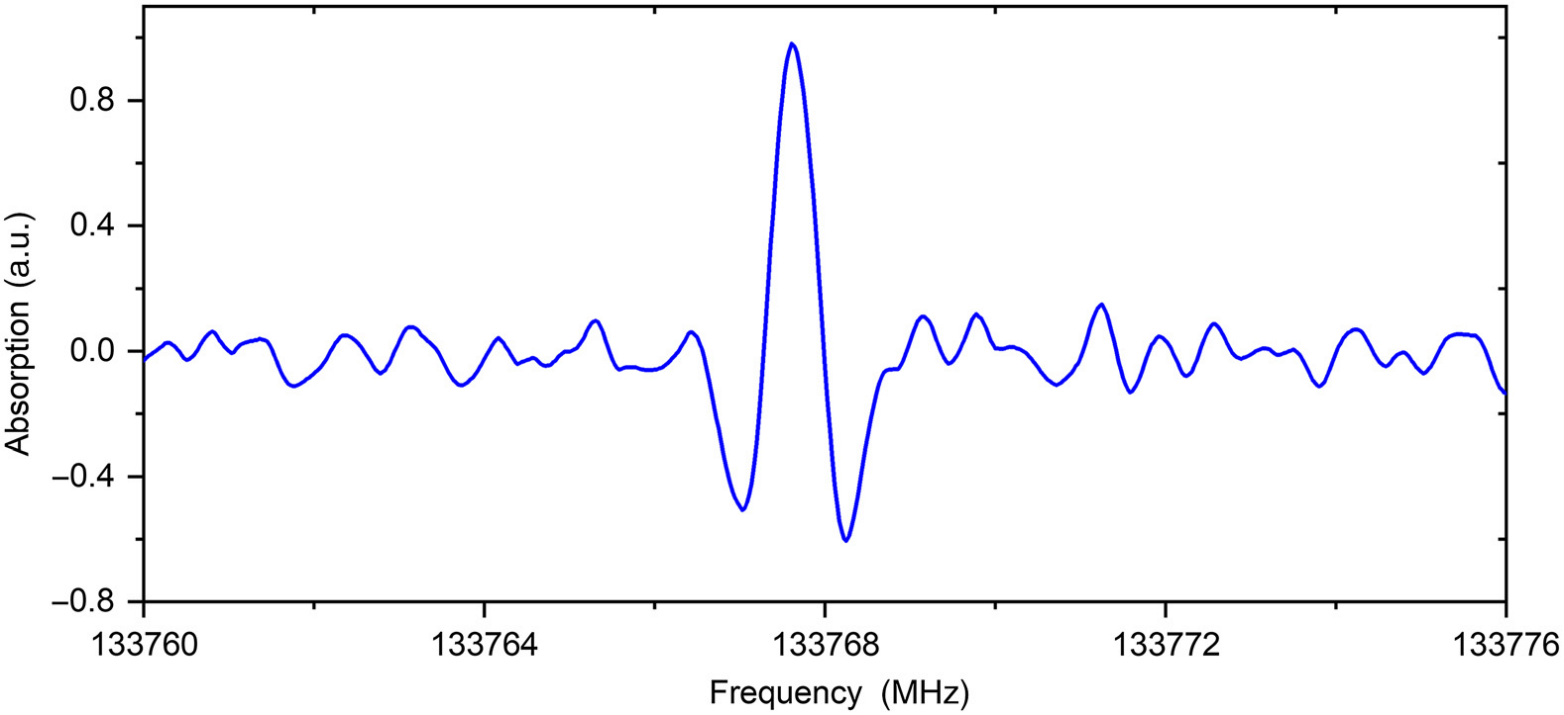
The record of formic acid absorption line (f=133.7671873  GHz, lgI=−3.8183 (6 0 6 ←5 0 5) in the sample of diabetic rats’ kidneys tissue.

**Fig. 3:**
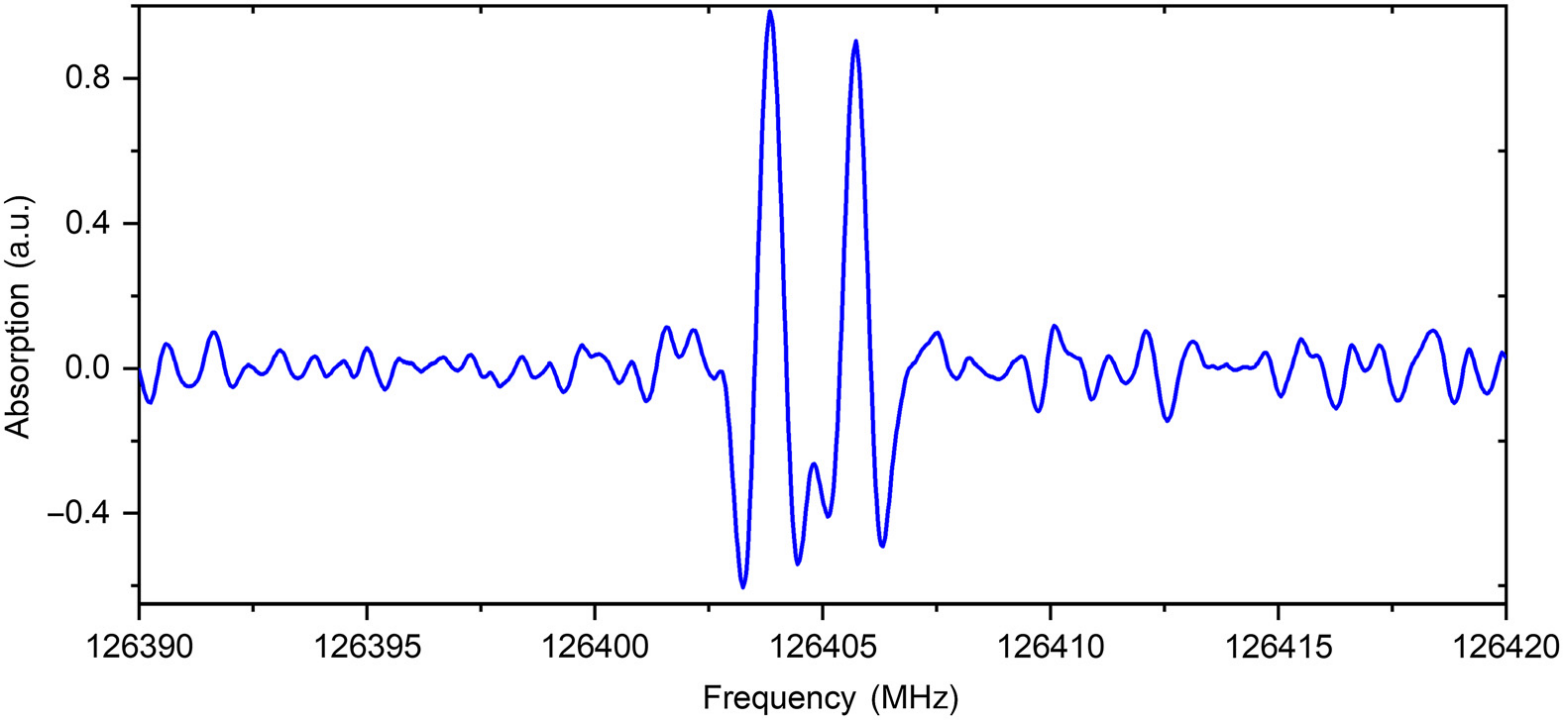
The record of methanethiol absorption lines ($f_{1}=126.4038344\;\mathrm{GHz}$, $lgI_{1}=-4.4357$, 5 0 5 1 ← 4 0 4 1 v=0 to 2 and $f_{2}=126.4056761\;\mathrm{GHz}$, $lgI_{2}=-4.4339$, 5 0 5 0 ← 4 0 4 0 v=0 to 2) in the sample of diabetic rats’ kidneys tissue.

The vapors samples of dried pellets of healthy and diabetic rats’ kidney tissues were studied. The vapors of kidney tissue of healthy rat contain carbonyl sulfide (OCS), sulfur dioxide (${\mathrm{SO}}_{2}$), formic acid (HCOOH), isocyanic acid (HNCO), ethyl formate ($C_{2}H_{5}\mathrm{OCHO}$), and propylene glycol (${\mathrm{CH}}_{2}(\mathrm{OH})\mathrm{CH}(\mathrm{OH}){\mathrm{CH}}_{3}$). The vapor content of diabetic rat kidney tissue was differed from the healthy rat by the presence of not only carbonyl sulfide (OCS) but also its isotopologue OCS-34. The acetaldehyde (${\mathrm{CH}}_{3}\mathrm{CHO}$), methanethiol (${\mathrm{CH}}_{3}\mathrm{SH}$), ethylene sulfide ($C_{2}H_{4}S$), methanol (${\mathrm{CH}}_{3}\mathrm{OH}$), and glycolaldehyde (${\mathrm{CH}}_{2}(\mathrm{OH})\mathrm{CHO}$) were also detected in the vapors of pellet of diabetic rat kidney tissue (see Fig. 2 and Fig. 3).

The presence of sulfur compounds, such as methanethiol, may be specified by thermal decomposition of sulfur amino acids (methionine, cysteine, and cystine) in the blood protein composition. Methionine is an essential amino acid that is not synthesized in human organism. It is obtained from the food and is a source of sulfur for cysteine biosynthesis. When sulfur amino acids were heated up to temperatures of about 240°C, methanethiol and ethylene sulfide (methionine decomposition) were detected in the mass spectrum of these substances.^20^

## 4 Conclusion

The molecular content of human and rat blood plasma and rat kidney tissue during thermal decomposition of the samples was studied with using the THz high-resolution spectrometer. The method of preparing the biological samples as pellets was presented. This method gives the possibility of storing and transportation of samples.

The high-resolution gas spectroscopy allows to determine the content of multicomponent gas mixtures of any origin, including biological with trace concentrations of gas components.

Acetone was detected in diabetic blood plasma samples. Information about the difference of content of diabetic and healthy blood plasma of men and rats and diabetic and healthy rat kidney tissues was obtained from absorption rotational spectra of vapors. The use of THz high-resolution spectroscopy provides a qualitative analysis of component content of samples of vapors of blood and kidney tissues pellets. The possibility of carrying out the quantitative estimations of concentrations of important diabetes gas-markers depending on the state of patients should be studied in further investigations.
